# Mortality and One-Year Functional Outcome in Elderly and Very Old Patients with Severe Traumatic Brain Injuries: Observed and Predicted

**DOI:** 10.1155/2015/845491

**Published:** 2015-11-24

**Authors:** Cecilie Røe, Toril Skandsen, Unn Manskow, Tiina Ader, Audny Anke

**Affiliations:** ^1^Department of Physical Medicine and Rehabilitation, Oslo University Hospital, Ulleval, Oslo, Norway; ^2^Research Centre for Habilitation and Rehabilitation Models and Services (CHARM), Faculty of Medicine, University of Oslo, Oslo, Norway; ^3^Department of Neuroscience, Faculty of Medicine, Norwegian University of Science and Technology (NTNU), Trondheim, Norway; ^4^Department of Physical Medicine and Rehabilitation, St. Olav's Hospital, Trondheim University Hospital, Trondheim, Norway; ^5^Faculty of Health Sciences, Institute of Clinical Medicine, University of Tromso, Norway; ^6^Department of Neurosurgery, University Hospital of North Norway, Tromso, Norway; ^7^Department of Physical Medicine and Rehabilitation, Haukeland University Hospital, Bergen, Norway; ^8^Department of Rehabilitation, University Hospital of North Norway, Tromso, Norway

## Abstract

The aim of the present study was to evaluate mortality and functional outcome in old and very old patients with severe traumatic brain injury (TBI) and compare to the predicted outcome according to the internet based CRASH (Corticosteroid Randomization After Significant Head injury) model based prediction, from the Medical Research Council (MRC).* Methods.* Prospective, national multicenter study including patients with severe TBI ≥65 years. Predicted mortality and outcome were calculated based on clinical information (CRASH basic) (age, GCS score, and pupil reactivity to light), as well as with additional CT findings (CRASH CT). Observed 14-day mortality and favorable/unfavorable outcome according to the Glasgow Outcome Scale at one year was compared to the predicted outcome according to the CRASH models.* Results.* 97 patients, mean age 75 (SD 7) years, 64% men, were included. Two patients were lost to follow-up; 48 died within 14 days. The predicted versus the observed odds ratio (OR) for mortality was 2.65. Unfavorable outcome (GOSE < 5) was observed at one year follow-up in 72% of patients. The CRASH models predicted unfavorable outcome in all patients.* Conclusion.* The CRASH model overestimated mortality and unfavorable outcome in old and very old Norwegian patients with severe TBI.

## 1. Introduction

Traumatic brain injury (TBI) is a major health problem, with high mortality in severe TBI [1]. For survivors, the injury may cause long-standing deficits that interfere with independent living, reduced levels of functioning and restrictions on activities [2]. The incidence of TBI among the elderly is increasing, posing a significant challenge on health care services in this group [3].

Mortality is particularly high among elderly patients [4]. A review of the literature indicated an overall mortality of 65% in severe TBI among patients above 60 years old [5]. The mortality was nearly twice as high among very old patients (≥75 years), compared to patients between 65 and 74 years. Long-term outcome is also assumed to be worse in the elderly [6]. This may be attributed to the consequences of biological ageing as well as chronic disease prevalence [7], thus rendering the elderly more prone to complications [8]. Assuming a poor prognosis may also influence the treatment strategies applied in older patients [9] and subsequently results in a self-fulfilling prophecy regarding outcome. One should keep in mind that even old subject with very severe TBI admitted with Glasgow Come Scale scores between three and four may have a favorable outcome [10]. In addition, older age (>65 years) has even been shown to predict better long-term life satisfaction [11]. The progress in intensive care and neurosurgical options increase the possibilities for treatment and survival [12]. Such treatment is expensive [13], and it has been argued that clinicians treating these patients need prognostic models guiding their treatment choices [14], and the elderly group should be no exception.

Determining the prognosis after TBI is challenging, in particular when it comes to long-term functional consequences [15]. Large samples covering the entire specter of individual and medical variations are needed [16]. The Medical Research Council (MRC) CRASH (Corticosteroid Randomization after Significant Head Injury) trial is the largest clinical trial conducted in patients with traumatic brain injury [17]. A web-based prognostic calculator for mortality and 6-month outcome is developed based on these data, available for clinical use [18]. The Scandinavian countries are characterized by high income, equal access to health and social care services, and long life expectancy (http://www.ssb.no/). Even though none of the Scandinavian countries were included in the trial, the CRASH algorithm provides the option of high income country in the calculation. The data included in CRASH model are routinely documented in the Norwegian trauma centers, and the specification of the older subpopulation in this database provided the rationale for choosing this model.

Hence, the aim of the present study was to evaluate the mortality and functional outcome in old and very old patients with severe TBI and compare the observed mortality and outcome to the predicted outcome according to the CRASH models. We also aimed to evaluate if more detailed descriptions of CT scans improved the prognostic accuracy and to which extent there were differences in the old and very old patients.

## 2. Material and Methods

### 2.1. Design and Study Region

This project is part of a prospective, multicenter, cohort study, comprising patients admitted with severe TBI to the regional hospitals in all four health regions in Norway during 2009 and 2010. Norway consists of a land area of 323 758 km^2^ and an adult population (aged ≥16 years) of 3.8 million (Statistics Norway). The Norwegian hospital structure includes local hospitals that serve small areas and regional trauma centres located in university hospitals that serve the local hospitals in the region.

### 2.2. Inclusion

In the current project, Norwegian residents ≥65 years of age who were admitted to their regional trauma centers within 72 hours of a severe TBI were included in the present part of the study. Severe TBI was defined by ICD 10 criteria (S06.1–S06.9) and a Glasgow Coma Scale (GCS) score between 3 and 8 within the first 24 hours after injury. The regional trauma centers were the University Hospital of North Norway for the northern region, St. Olav's Hospital Trondheim University Hospital for the middle region, and Oslo University Hospital for the southeastern region. In the western part of the country, patients are equally distributed between Haukeland University Hospital and Stavanger University Hospital. Unfortunately, Stavanger University Hospital was not able to participate. Exclusion criteria were chronic subdural hematomas (SDH), preinjury cognitive disability, and severe psychiatric disease or drug abuse. This study was approved by the regional Committee for Medical Research Ethics, Southeast Norway (S-08378a, 2008/10441).

### 2.3. Data Collection

Data registration was based on a standardized review of hospital journals (paper and electronic records), CT scans, and data from the trauma registries. Follow-up at 12 months included clinical examination and collecting supplementary information regarding demographic data and functional levels which was collected from patients and their relatives.

### 2.4. Demographic, Medical, and Injury Characteristics

The causes of injury were classified as transport accidents, falls, violence, or other causes including sports injuries. Transport with intermediate stays at local hospitals prior to admittance to the trauma center was recorded as yes or no.

The comorbidity status was classified as none or having a medical disease at the time of injury. Anticoagulant status was defined by the use of warfarin or platelet inhibitors. The influence of alcohol or other substances at admission was categorized as yes or no, based on clinical judgment and blood or urine analysis, when available.

### 2.5. Injury Severity and Surgical Treatment

The GCS score was assessed at the accident scene and at hospital admittance. The lowest GCS score recorded within the first 24 hours is presented and used in the analysis. Dilation of the pupils was recorded based on the prehospital charts and at admission and collapsed into no, one, or two dilated pupils. The Injury Severity Score (ISS) version 2008 was applied to indicate overall trauma severity. ISS of 9 or more added to the head injury abbreviated injury score (AIS) was considered as major extracranial trauma. The CT findings were described by a neurosurgeon or a radiologist. The presence of petechial hemorrhages, hematomas (epidural, subdural, and subarachnoid), obliteration of the third ventricle and basal cisternae, and midline shift were defined. Intracranial surgery was recorded, including ICP monitoring, cerebrospinal fluid (CSF drainage), craniotomy, and craniectomy. Information of craniotomy was used in order to evaluate whether the patient had a nonevacuated hematoma.

### 2.6. Outcome

Mortality within the first 14 days was assessed.

The TBI related, global functional outcome at 12 months was evaluated in survivors by structured interview using the Glasgow Outcome Scale Extended (GOSE) [19, 20]. GOSE is scored on an ordinal score from 1 (dead) to 8 (no functional sequel from TBI). Outcome was categorized as unfavorable (GOSE scores 1 to 4) and favorable (GOSE scores 5 to 8).

Furthermore, living situation (home, service home or institution, need for assistance (several times a day, daily, regularly, and never), and driving a car (yes/no)) was recorded at 12-month follow-up. Life satisfaction was measured with 1 global item: “Overall, how satisfied are you with your life situation now?” The item was rated on a 5-point ordinal scale: 1 (very dissatisfied), 2 (dissatisfied), 3 (neither satisfied nor dissatisfied), 4 (satisfied), or 5 (very satisfied) [21].

### 2.7. CRASH Prediction

Based on the MRC CRASH trial prognostic models including country, age, GCS score, pupil reactivity to light (both, one, or none) were developed and are available at the web (http://www.trialscoordinatingcentre.lshtm.ac.uk/Risk%20calculator/index.html). A second model also including CT characteristics (petechial hemorrhages, hematomas (epidural, subdural, and subarachnoid), obliteration of the third ventricle and basal cisterna, and midline shift) is also available. The CRASH models predict 14-day mortality and unfavorable outcome after 6 months. Death and GOSE score below 5 are assigned as unfavorable outcome.

### 2.8. Data Analysis and Statistics

The predicted mortality within 14 days and unfavorable outcome were calculated according to the web-based CRASH basic and CRASH CT prediction models for each patient. Dilated pupils were considered nonreactive to light and entered together with age and the lowest GCS within 24 hours. The percentage of patients predicted to be dead or having an unfavorable outcome was reported. Odds ratio (OR) with confidence intervals (CI) was used to calculate differences between the observed and CRASH based estimated 14-day mortality as well as differences in old and very old patients. In addition, the OR for predicted unfavorable 6-month outcome versus observed unfavorable outcome at 12 months was calculated. The chi square (*χ* ^2^) test for contingency tables was used to detect associations between categorical independent variables, including differences in outcome between the old (65–74 years) and very old patients (≥74 years). The analysis was conducted in IBM SPSS Statistics V21. A statistical significance level of 0.05 was adopted.

## 3. Results

A total of 97 patients with mean age 75 (SD 7), 62 men and 35 women, were included. Hence, 52% of the patients were 75 years or older, with three persons over 90 years at the time of injury. The traditional predominance of males is less prominent among the oldest patients (Table 1). The most frequent mechanism of injury was fall (84%), followed by transport accidents (12%) and violence 1%, and 3% with other injury mechanisms in the cohort without statistically significant differences between the age categories. About half of the patients were transported via local hospitals. Prevalence of comorbidity and use of anticoagulation therapy increased with age (Table 1). Nonevacuated hematomas were more frequent in the very old group whereas other injury characteristics were rather similar across age (Table 2).

### 3.1. Mortality and One-Year Outcome

**Figure 1:**
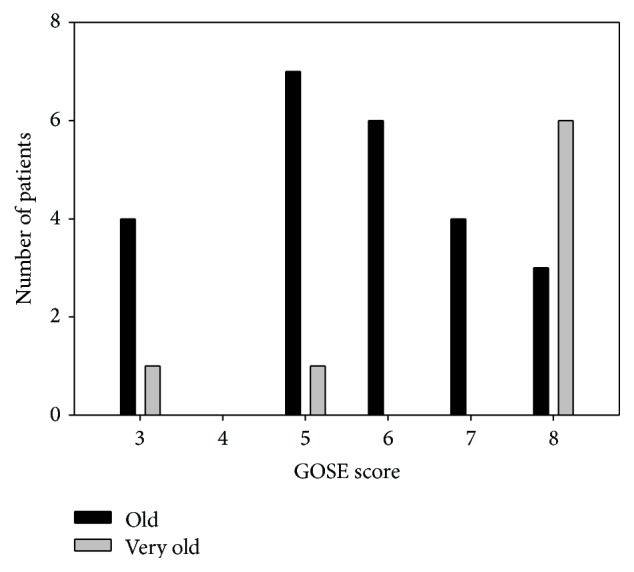
Distribution of GOSE score for the surviving old (black bars) and very old (grey bars) patients at 12-month follow-up.

48 patients died within 14 days; additionally, 12 patients died before 3 months and three patients before 12-month follow-up. Two patients were lost to follow-up at 12 months. Hence, 14-day overall mortality was 50% in the present study. The observed mortality was significantly higher in the very old compared to the old group (OR = 3.16, *p* = 0.006). At one-year follow-up unfavorable outcome was observed in 72% of the patients. The observed outcome was significantly more favorable in the old compared to the very old patients (OR = 4.84, CI 1.80 to 12.99, *p* = 0.002). Although only eight very old patients survived, it is worth noting that the functional level of the majority of these patients was not heavily influenced as evaluated by GOSE (Figure 1). Furthermore, the majority was still living at home, with low level of assistance, and reported an overall high level of satisfaction (Table 3).

### 3.2. CRASH Predicted Compared to Observed Clinical Outcome

The predicted mortality according to the CRASH basic model was significantly higher than the observed (OR = 2.65, CI 1.46 to 4.40) (*p* = 0.001), with particular discrepancy in the old patients (Table 4). Adding CT findings to the CRASH basic prediction model rendered an even higher OR of predicted versus observed mortality (OR = 6.60, CI 3.25 to 13.37) (*p* < 0.001). Unfavorable outcome (GOSE < 5) was observed at one-year follow-up in 72% of patients whereas the CRASH basic and CRASH CT models predicted unfavorable outcome in all patients (Table 4).

## 4. Discussion

The present study underscores the impact of severe TBI in elderly patients. Half of the patients died within 14 days and, additionally, 12% died within one-year follow-up. On the other hand, the outcome was strikingly better than the predicted outcome according to the CRASH algorithm. The reported level of life satisfaction in survivors was also rather high.

CRASH and the International Mission for Prognosis and Analysis of Clinical Trial in TBI (IMPACT) represent the largest datasets available for predicting outcome after TBI [17, 22]. Forty-eight countries participated and models for high compared to low income countries are developed and validated externally [18, 23]. The IMPACT differs from the CRASH model by using motor score instead of GCS score. In addition, hypoxia and hypotension are included in the prediction model. In the present Norwegian multicenter study we had complete dataset for GCS whereas only some centers recorded motor score. Focusing on the elderly rendered also the possibility to run statistical comparisons with the 902 patients above 64 years encountered in the CRASH study [17].

The efficacy of different treatment options in severe TBI varies according to injury and patient characteristics [15]. One of the best predictors is the pattern of recovery after TBI, particularly regarding level of consciousness [24]. However, in the acute phase of severe TBI decisions about surgical and neurointensive treatment have to be made promptly. Given the complexity of severe TBI, statistical models that combine data from patients to predict outcome are likely to be more accurate than simple clinical predictions [16]. Recent study from the Oslo University Hospital (OUH) on external validation of a prognostic model for early mortality after TBI developed at the University of Southern California (USC) showed that the USC model overestimated mortality in OUH population [25]. Similarly, in the present study of elderly Norwegian patients sustaining severe TBI, the agreement between the predicted and the observed 14-day mortality was low. Although the mortality in the elderly was high compared to the overall TBI mortality in Norway [26], these elderly patients had a substantially higher survival rate than predicted. One of the reasons for this could be related to general and neurotrauma-targeted improvements in our trauma services since late 2004 such as “a formalized trauma service, damage control resuscitation protocols, structured training, increased helicopter transfer capacity, consultant-based neurosurgical assessment, a doubling of emergency neurosurgical procedures, and improved neurointensive care” [27]. In general, the acute hospital care and postacute community-based health care in Norway are of high standard and are free for all citizens regardless of income. Hence, all of the patients in this study have had access to appropriate health care services. This may not be the case for all people in the sample of the elderly in the CRASH study, not even within the heterogeneous group of high income countries. For instance, recent study from the US reported that elderly patients with TBI who were designated as self-paying showed higher odds of death [28]. High general life expectancy in Norway could also be one possible cause of these results. The life expectancy in 2009 was 83 years for women and 79 years for men (http://www.ssb.no/), which is among the top ten countries in the world (http://www.globalis.no/). According to this, a generally better health among the elderly in Norway compared to other countries may also be a possible reason. However, the majority of the West European countries have life expectancies close to 80 years. Regarding quality of life, Norway is ranked number one by the United Nations (UN) statistics, which could be taken into account for a better health status in Norway than in many other European countries. Included in the UN statistics is also the economic situation which takes into account the level of health care service (Discovery News, February 11, 2013, http://www.discovery.com/). The survival rate for major trauma in the acute phase is also high in Norway [29].

Misclassification of predictors or outcome may also contribute to the discrepancy between the observed and predicted outcome. The mortality outcome was based on the hospital records supplemented with updated Norwegian death statistics, and we deem the possibility for misclassification as very low. In the Norwegian multicenter study functional outcome was evaluated by GOSE, whereas Glasgow Outcome Scale (GOS) was used in the CRASH study. GOSE subdivide the severe and moderate disability, but the threshold for favorable versus unfavorable outcome should be equal in GOSE and GOS. We can only speculate regarding whether assessments in the present study may have influenced outcome. However, the better performance of models predicting mortality compared to functional outcome is in accordance with the recent results of Majdan et al. [30]. Regarding the included clinical predictors misclassification of pupils not reacting to light is a possible bias. This parameter may also be prone to transport distance to hospital and present more frequently at admission with respect to comparable intracranial injuries when the transport distance is long.

The evaluation of substantial extracranial injuries was based on ISS of 9 or more caused by other traumas than the head. In CRASH model additional major trauma was defined as an injury which alone would be reason to hospital admission, and one could discuss if ISS is a very low level and thus contributed to the differences in observed and predicted outcomes.

One would also assume that including more information in the model, that is, CT findings, would improve the agreement. However, the actual agreement declined further when including CT findings. In the present study the CT with the most extensive injuries were chosen. The elderly have a higher frequency of hematomas [8], and the number of nonevacuated hematomas was high. These findings are generally associated with a poor prognosis [31]. We can only speculate whether the subdural and other hematomas in the elderly have a slightly different course and impact on outcome compared to younger patients, which are not fully accounted for in the model. Possibly the general atrophy in the old brain renders more space for expansion compared to the younger patients CT [32]. However, misclassifications of the CT parameters in the present study cannot be excluded, even though experiences radiologists evaluated the CT scans.

The CRASH model is developed to predict outcome after 6 months. The follow-up in the Norwegian national study was one year after injury. We cannot exclude that recovery from 6 to 12 months contributed to the much better outcome observed than the outcome predicted by the CRASH algorithm [33]. However, the effect of age will also increase with time to follow-up counteracting the effect of recovery in this elderly group. The CRASH algorithm includes the same predictors for 14-day mortality and for 6-month outcome. Gaetani et al. however documented that tSAH in the elderly influenced mortality but not long-term outcome [34]. It is also worth noting that, of the 32 survivors at 12-month follow-up, only five had a GOSE score below five. These results are in agreement with Flanagan et al. [35], also emphasizing a more optimistic prognosis in the elderly than previously assumed. The feared shifting from mortality to vegetative or reduced consciousness state over time does not seem to be supported [36]. Further, the rationale behind the algorithm could be questioned, as unfavorable outcome equals dead with a disability level that implies a person with a functional level with some dependency of others, but the person can be alone for at least 8 hours. This is also emphasized in recent external validations using ordinal outcome of GOSE instead of dichotomizing in favorable and unfavorable outcome [37].

In conclusion, the present more favorable observed compared to predicted outcome and the reported general life satisfaction in the survivors are worth considering when deciding on neurosurgical treatment as well as rehabilitation in elderly TBI patients.

## Figures and Tables

**Table 1:** Demographic characteristics and injury mechanisms of the old (65–74 years) and very old (75–92 years) are presented.

	Old (65–74 y, *n* = 46)	Very old (≥75 y, *n* = 51)	Chi square	*p* value
Male	74% (*n* = 34)	55% (*n* = 28)	3.79	0.05
Married/cohabitant	67% (*n* = 31)	53% (*n* = 27)	2.10	0.15
Comorbidity	76% (*n* = 35)	88% (*n* = 45)	2.47	0.12
Anticog. medication^¤^	46% (*n* = 21)	73% (*n* = 37)	7.28	0.007
Injury mechanism				
Fall	78% (*n* = 36)	88% (*n* = 45)	4.76	0.19
Transport	13% (*n* = 6)	12% (*n* = 6)		
Violence	2% (*n* = 1)	0%		
Sports/other	7% (*n* = 3)	0%		
Transport via local hospital	57% (*n* = 26)	45% (*n* = 23)	1.26	0.26

y = years.

^¤^Anticoagulation and platelet inhibitors.

**Table 2:** Distribution of injury severity and CT based findings in the old and very old patients.

	Old (65–74 y, *n* = 46)	Very old (≥75 y, *n* = 51)
GCS (median, IQR**)**	6 (4–8)	4 (3–7)
Dilated pupils		
Both	15% (*n* = 7)	24% (*n* = 12)
One	22% (*n* = 10)	33% (*n* = 17)
None	63% (*n* = 29)	43% (*n* = 22)
Petechial hemorrhage	70% (*n* = 32)	71% (*n* = 36)
Obliteration of third ventricle/basal cisternae	67% (*n* = 31)	75% (*n* = 38)
SAH	76% (*n* = 35)	59% (*n* = 30)
Midline shift	46% (*n* = 21)	62% (*n* = 32)
Nonevacuated hematomas	37% (*n* = 17)	51% (*n* = 26)

**Table 3:** Characteristics of the surviving old and very old patients at one-year follow-up.

	Old (*n* = 24) (65–74 y, *n* = 24)	Very old (≥75 y, *n* = 8)
Living situation		
At home	20	7
Service home	2	0
Institution	2	1
Assistance at home		
None	14	5
Regularly	5	0
Daily	1	2
Several times a day	4	1
Driving a car	7	4
Satisfaction (mean, SD)	4.10, 0.83	4.25, 1.04

**Table 4:** Observed 14-day mortality and unfavorable outcome of 12 months and CRASH predicted 14-day mortality and 6-month outcome based on clinical (CRASH basic) and combined clinical and CT based information (CRASH CT). Results are shown for all patients ≥ 65 years, the old group (65–74 years), and the very old group (**≥**75 years).

	Observed	Predicted (CRASH basic)	Predicted (CRASH CT)
≥65 years (*n* = 97)			
Mortality	50%	64 (56–71)%	81 (73–87)%
Unfavorable outcome	72%	90 (86–92)%	95 (91–96)%
65 to 74 years (*n* = 46)			
Mortality	35%	54 (45–56)%	74 (64–81)%
Unfavorable outcome	44%	85 (80–87)%	92 (88–95)%
≥75 years (*n* = 51)			
Mortality	63%	73 (65–80)%	88 (81–92)%
Unfavorable outcome	86%	93 (91–95)%	97 (94–98)%

Unfavorable outcome is defined as dead and GOSE score <5.

## References

[1] Masson F., Thicoipe M., Aye P. (2001). Epidemiology of severe brain injuries: a prospective population-based study. *Journal of Trauma—Injury, Infection and Critical Care*.

[2] Andelic N., Hammergren N., Bautz-Holter E., Sveen U., Brunborg C., Røe C. (2009). Functional outcome and health-related quality of life 10 years after moderate-to-severe traumatic brain injury. *Acta Neurologica Scandinavica*.

[3] Andelic N., Sigurdardottir S., Brunborg C., Roe C. (2008). Incidence of hospital-treated traumatic brain injury in the Oslo population. *Neuroepidemiology*.

[4] Utomo W. K., Gabbe B. J., Simpson P. M., Cameron P. A. (2009). Predictors of in-hospital mortality and 6-month functional outcomes in older adults after moderate to severe traumatic brain injury. *Injury*.

[5] McIntyre A., Mehta S., Aubut J., Dijkers M., Teasell R. W. (2013). Mortality among older adults after a traumatic brain injury: a meta-analysis. *Brain Injury*.

[6] de Guise E., LeBlanc J., Dagher J. (2015). Traumatic brain injury in the elderly: a level 1 trauma centre study. *Brain Injury*.

[7] McIntyre A., Mehta S., Janzen S., Aubut J., Teasell R. W. (2013). A meta-analysis of functional outcome among older adults with traumatic brain injury. *NeuroRehabilitation*.

[8] Røe C., Skandsen T., Anke A. (2013). Severe traumatic brain injury in norway: impact of age on outcome. *Journal of Rehabilitation Medicine*.

[9] Munro P. T., Smith R. D., Parke T. R. J. (2002). Effect of patients' age on management of acute intracranial haematoma: prospective national study. *British Medical Journal*.

[10] Brazinova A., Mauritz W., Leitgeb J. (2010). Outcomes of patients with severe traumatic brain injury who have Glasgow Coma Scale scores of 3 or 4 and are over 65 years old. *Journal of Neurotrauma*.

[11] Anke A., Andelic N., Skandsen T. (2015). Functional recovery and life satisfaction in the first year after severe traumatic brain injury: a prospective multicenter study of a norwegian national cohort. *Journal of Head Trauma Rehabilitation*.

[12] Sundstrøm T., Sollid S., Wester K. (2005). Deaths from traumatic brain injury in the Nordic countries, 1987–2000. *Tidsskrift for den Norske Laegeforening*.

[13] Olesen J., Gustavsson A., Svensson M., Wittchen H.-U., Jönsson B. (2012). The economic cost of brain disorders in Europe. *European Journal of Neurology*.

[14] Perel P., Edwards P., Wentz R., Roberts I. (2006). Systematic review of prognostic models in traumatic brain injury. *BMC Medical Informatics & Decision Making*.

[15] Fleminger S., Ponsford J. (2005). Long term outcome after traumatic brain injury. *British Medical Journal*.

[16] Lee K. L., Pryor D. B., Harrell F. E. (1986). Predicting outcome in coronary disease statistical models versus expert clinicians. *The American Journal of Medicine*.

[17] Roberts I., Yates D., Sandercock P. (2004). Effect of intravenous corticosteroids on death within 14 days in 10008 adults with clinically significant head injury (MRC CRASH trial): randomised placebo-controlled trial. *The Lancet*.

[18] Perel P. A., Arango M., Clayton T., et al (2008). Predicting outcome after traumatic brain injury: practical prognostic models based on large cohort of international patients. *British Medical Journal*.

[19] Jennett B., Bond M. (1975). Assessment of outcome after severe brain damage. *The Lancet*.

[20] Wilson J. T. L., Pettigrew L. E. L., Teasdale G. M. (1998). Structured interviews for the Glasgow outcome scale and the extended Glasgow outcome scale: guidelines for their use. *Journal of Neurotrauma*.

[21] Kolakowsky-Hayner S. A., Miner K. D., Kreutzer J. S. (2001). Long-term life quality and family needs after traumatic brain injury. *Journal of Head Trauma Rehabilitation*.

[22] Marmarou A., Lu J., Butcher I. (2007). IMPACT database of traumatic brain injury: design and description. *Journal of Neurotrauma*.

[23] Edwards P., Arango M., Balica L. (2005). Final results of MRC CRASH, a randomised placebo-controlled trial of intravenous corticosteroid in adults with head injury-outcomes at 6 months. *The Lancet*.

[24] Eastvold A. D., Walker W. C., Curtiss G., Schwab K., Vanderploeg R. D. (2013). The differential contributions of posttraumatic amnesia duration and time since injury in prediction of functional outcomes following moderate-to-severe traumatic brain injury. *Journal of Head Trauma Rehabilitation*.

[25] Rønning P. A., Pedersen T., Skaga N. O., Helseth E., Langmoen I. A., Stavem K. (2011). External validation of a prognostic model for early mortality after traumatic brain injury. *Journal of Trauma—Injury Infection & Critical Care*.

[26] Andelic N., Anke A., Skandsen T. (2012). Incidence of hospital-admitted severe traumatic brain injury and in-hospital fatality in Norway: a national cohort study. *Neuroepidemiology*.

[27] Søvik S., Skaga N. O., Hanoa R., Eken T. (2014). Sudden survival improvement in critical neurotrauma: an exploratory analysis using a stratified statistical process control technique. *Injury*.

[28] Haring R. S., Narang K., Canner J. K. (2015). Traumatic brain injury in the elderly: morbidity and mortality trends and risk factors. *Journal of Surgical Research*.

[29] Kristiansen T., Lossius H. M., Søreide K., Steen P. A., Gaarder C., Næss P. A. (2011). Patients referred to a Norwegian trauma centre: effect of transfer distance on injury patterns, use of resources and outcomes. *Journal of Trauma Management and Outcomes*.

[30] Majdan M., Lingsma H. F., Nieboer D., Mauritz W., Rusnak M., Steyerberg E. W. (2014). Performance of IMPACT, CRASH and Nijmegen models in predicting six month outcome of patients with severe or moderate TBI: an external validation study. *Scandinavian Journal of Trauma, Resuscitation and Emergency Medicine*.

[31] Leitgeb J., Mauritz W., Brazinova A. (2012). Outcome after severe brain trauma due to acute subdural hematoma: clinical article. *Journal of Neurosurgery*.

[32] Cardoso E. R., Del Bigio M. R., Schroeder G. (1989). Age-dependent changes of cerebral ventricular size. Part I: review of intracranial fluid collections. *Acta Neurochirurgica*.

[33] Corral L., Ventura J. L., Herrero J. I. (2007). Improvement in GOS and GOSE scores 6 and 12 months after severe traumatic brain injury. *Brain Injury*.

[34] Gaetani P., Revay M., Sciacca S. (2012). Traumatic brain injury in the elderly: considerations in a series of 103 patients older than 70. *Journal of Neurosurgical Sciences*.

[35] Flanagan S. R., Hibbard M. R., Gordon W. A. (2005). The impact of age on traumatic brain injury. *Physical Medicine and Rehabilitation Clinics of North America*.

[36] Patel H. C., Bouamra O., Woodford M., Yates D. W., Lecky F. E. (2010). Clinical article: mortality associated with severe head injury in the elderly. *Acta Neurochirurgica*.

[37] Lingsma H. F., Yue J. K., Maas A. I. R. (2015). Outcome prediction after mild and complicated mild traumatic brain injury: external validation of existing models and identification of new predictors using the TRACK-TBI pilot study. *Journal of Neurotrauma*.

